# The potential of molecular testing and mutation analysis for detecting isoniazid and rifampicin-resistant *Mycobacterium tuberculosis* in Thailand

**DOI:** 10.1590/S1678-9946202668040

**Published:** 2026-07-03

**Authors:** Janisara Rudeeaneksin, Benjawan Phetsuksiri, Sopa Srisungngam, Supranee Bunchoo, Payu Bhakdeenuan, Wiphat Klayut

**Affiliations:** 1 Ministry of Public Health, National Institute of Health, Department of Medical Sciences, Nonthaburi, Thailand; 2 Ministry of Public Health, Medical Sciences Technical Office, Department of Medical Sciences, Nonthaburi, Thailand

**Keywords:** Mycobacterium tuberculosis, Mutations, Isoniazid, Rifampicin, Anyplex

## Abstract

Drug-resistant tuberculosis (TB) poses a significant threat, and drug-susceptibility testing (DST) is essential for effective TB treatment and control. Genotypic DST has been used for the early detection of drug-resistant TB, and the detection of isoniazid (INH) resistance is increasingly important. This study aims to determine the potential of genotypic DST using the Anyplex^TM^ II MTB/MDR real-time polymerase chain reaction (PCR) kit and analyze the mutations associated with INH and rifampicin (RIF) resistance in *Mycobacterium tuberculosis* (MTB). DST and DNA sequencing of the *katG, inhA*, and *rpoB* genes were performed in 146 MTB isolates. Compared with phenotypic DST, the Anyplex real-time PCR assay showed the sensitivity of 84.0%, 83.3%, 85.7%, and 94.6%; and the specificity of 96.9%, 96.4%, 98.2%, and 92.3% for the detection of INH resistance, RIF resistance, multidrug-resistant TB (MDR-TB), and drug-susceptible MTB, respectively. Substantial agreement with DNA sequencing was observed. For INH resistance, 91.8% had mutations in the *katG* or *inhA*; the *katG* gene accounted for 71.8%, and the *inhA* promoter region accounted for 20.0%. S315T was predominant in the *katG* mutation, and only C(-15)T was present in the *inhA* mutation. For RIF resistance, 95.1% harbored the *rpoB* mutation, with S531L and D516V being the two most common. The association of mutations with INH and RIF resistance was high. The study highlighted the significance of data on the genetic determinants of INH and RIF resistance, and suggested the potential of using genotypic DST, such as the Anyplex II MTB/MDR assay, in the country and similar settings.

## INTRODUCTION

Tuberculosis (TB), caused by *Mycobacterium tuberculosis* (MTB), remains the leading cause of morbidity and mortality from an infectious disease globally^1^ and drug-resistant TB (DR-TB) is a critical public health issue, particularly in developing countries, including Thailand. In 2024, the global TB burden was estimated at 10.7 million new cases, and 1.23 million deaths^1^, representing a decrease from 10.8 million cases estimated in 2023^2^, and 3% reduction compared with 1.27 million deaths^1^. Recent reductions in TB cases and deaths were reversed as a consequence of the COVID-19 pandemic, which substantially increased the number of undiagnosed TB cases^2^. The persistent threat of multidrug-resistant TB (MDR-TB), defined as resistance to at least isoniazid (INH) and rifampicin (RIF), the two most potent anti-TB drugs, has exacerbated the problem as it can lead to treatment failure and the spread of the disease. Several strategies to enhance diagnostic capabilities for DR-TB have been proposed^3^. MTB strains can develop mono-resistance to INH or RIF, as well as become MDR. Approximately 390,000 people developed MDR/RIF-resistant-TB (RR-TB) globally in 2024 (a decrease from 410,000 in 2023) and there were an estimated 1.5 million incident cases of INH resistance^1^. MDR-TB is more prevalent in Asia, especially Southeast Asia, compared to other continents^1^,^3^,^4^. Thailand is one of the 30 highest-TB burden countries with an estimated 146 cases per 100,000 in 2024^1^, and has shown a decline in TB incidence following the increase observed during the COVID-19 pandemic. Drug-susceptibility testing (DST), especially for INH and RIF, is imperative for effective TB treatment and control.

The primary mechanisms of drug resistance in MTB are mutations in genes encoding drug targets, in regulatory regions of these genes, or in genes involved in drug activation, affecting the efficacy of anti-TB drugs^4,5^. Mutations associated with INH resistance occur in many genes involved in multiple biosynthetic pathways, such as *katG, inhA*, *ahpC, kasA*, *ndh, iniABC*, *fadE, furA*, *Rv1592c*, and *Rv1772* ^6,7^. The *katG* mutations are the most prevalent, resulting in a wide range of moderate- to high-level resistance, and the most frequently observed *katG* mutation is S315T^7-9^, accounting for approximately 64% of all *katG* mutations worldwide^7^. In contrast, low-level resistance to INH is mainly caused by mutations in the promoter region of the *inhA*, primarily at positions −15 and −8^10,11^. The C−15T *inhA*-mutation is predominant and present on average of 19% of INH-resistant clinical isolates worldwide^7^. Less frequently, mutations in several other genes usually cause low-level INH resistance^6,12^. Altogether, resistance to INH is widespread and the most prevalent among drug-resistant TB^13^. For RIF resistance, about 95–98% of RIF-resistant strains have mutations in the *rpoB* gene, and more than 95% of these are located in the 81-base pair of the RIF Resistance-Determining Region (RRDR), known as a mutation hotspot region for RIF resistance^7,14,15^. The frequently mutated codons are 531, 526, and 516^12,14,16^. Although several studies have reported similar mutations associated with INH and RIF resistance, the frequency of each mutation may vary depending on MTB populations and their distribution across regions^7,16,17^.

The rapid and accurate detection of TB, followed by DST, at least for INH and RIF resistance, is essential for initiating appropriate treatment and controlling the transmission of TB and DR-TB. Culture-based phenotypic DST is the gold standard for diagnosing DR-TB. However, it is labor-intensive, requires complex biosafety facilities, and well-trained personnel. It takes a long time to produce results due to the slow growth rate of MTB, even with the faster automated culture methods, thereby delaying effective treatment and potentially increasing the incidence of MDR-TB^7,18^. In contrast, molecular or genotypic DST provides rapid results for the early detection of DR-TB by simultaneously detecting multiple frequent mutations that confer drug resistance^18-20^. Xpert MTB/RIF, an automated system based on real-time polymerase chain reaction (PCR), has been widely used for the rapid detection of MTB and RIF resistance. It can identify patients eligible for MDR-TB treatment based on evidence that most RR-TB cases are also resistant to INH, and that targeting *rpoB* is a sensitive approach for detecting RIF resistance and, consequently, MDR-TB^20,21^. In 2017, the World Health Organization (WHO) recommended a new regimen, consisting of RIF, ethambutol, pyrazinamide, and levofloxacin for six months, for INH-resistant TB. Consequently, early diagnosis of INH resistance is increasingly important, as well as MDR-TB and RR-TB^22,23^. Over the years, the Anyplex II MTB/MDR Detection (Seegene Inc., Republic of Korea) has been described for the rapid detection of INH- and RIF-resistant TB, including MDR-TB^24^. The assay is a semi-automated real-time PCR system, designed to simultaneously detect MTB and the frequent 25 mutations associated with phenotypic resistance to INH and RIF based on melting curve analysis. The assay has been used in Thailand and other countries, such as Mexico^25,26^. However, a limited number of studies have compared this assay to phenotypic DST as well as DNA sequencing, given that the frequency and distribution of mutations can impact DST by genotypic detection. This study aims to determine the potential of the Anyplex II MTB/MDR assay (hereafter referred to as Anyplex MTB/MDR) to identify INH- and RIF-resistant MTB by comparing it to phenotypic DST and DNA sequencing, and to investigate mutations in the *katG, inhA* (the promoter), and *rpoB* genes in MTB and their associations with INH and RIF resistance.

## MATERIALS AND METHODS

### MTB samples and microbiological processing

The study was carried out using MTB isolates obtained from the culture collection. All isolates used were sourced from pulmonary TB patients and all stored INH and/or RIF-resistant MTB samples were included in the study. Sample processing and phenotypic DST were performed previously as part of the routine laboratory testing for TB patient care. Briefly, sputum samples were processed for mycobacterial culture using the *N*-acetyl L-cysteine-sodium hydroxide (2% final concentration). The suspension of the sediment resuspended in 0.067 M phosphate buffer (pH 6.8) was finally inoculated on Lowenstein-Jensen (LJ) solid media for conventional culture, and inoculated into MGIT liquid media (Becton Dickinson, USA) for rapid mycobacterial growth detection by the BACTEC™ MGIT 960 System (Becton Dickinson). Positive cultures were confirmed for MTB using SD Bioline TB Ag rapid test, an immunochromatographic test according to the manufacturer's instructions (Standard Diagnostics, Inc., South Korea). Standard phenotypic DST for first-line drugs was performed using the MGIT 960 DST SIRE kit (Becton Dickinson) following the manufacturer's procedure. In this study, the retrievable isolates were sub-cultured on LJ slants and grown at 37 °C for use in the analyses.

### DNA extraction

DNA was extracted from MTB isolates using the DNA-extraction solution provided in the Anyplex MTB/MDR kit. In brief, a loop of MTB colonies was transferred into a 1.5 mL centrifuge tube containing 200 µL of DNA extraction solution containing beads. The samples were then boiled in a dry-heat block for 20 min, and centrifuged at 13,000 rpm for 5 min. The supernatant containing DNA was recovered and finally collected in new tubes for use as a DNA template.

### Genotypic DST by real-time multiplex PCR

All DNA samples were subjected to real-time multiplex PCR using the Anyplex MTB/MDR kit following the manufacturer's recommendation. The assay detected IS*6110* and MPT64 sequences for the existence of MTB, and mutations in the *katG, inhA*, and *rpoB* genes^24,27^. For INH resistance, detection targeted the *katG* mutations S315I, S315N, S315T (AGC>ACC), and S315T (AGC>ACA), as well as the *inhA* promoter −15T, −8C, and −8A, using a total of seven probes labeled with the fluorophore Cal Red 610^24,27^. For RIF resistance, the assay detected the most frequent mutations spanning codons 511−533 in the *rpoB* gene consisting of L511P, Q513K, Q513L, Q513P, 3-amino acid deletion in codons 513−516, D516V, D516Y, S522L, S522Q, H526C, H526D, H526L, H526N, H526R, H526Y, S531L, S531W, and L533P, using 18 probes labeled with the fluorophore HEX (hexachlorofluorescein). The change in the melting temperature (*Tm*) was considered an indicator of a mutation, and isolates for which the probe had a *Tm* other than that for *M. tuberculosis* H37Rv were considered to be resistant to RIF and/or INH^24^.

To amplify target sequences, each real-time PCR reaction was prepared in a total volume of 20 µL following the manufacturer's instructions. Briefly, 5 µL of DNA template obtained from each sample was added to 15 µL of the reaction mix, consisting each 5 µL of 4X Anyplex PCR Master mix, 4X Anyplex MTB/MDR TOM (oligo primers and probes mix) and RNase-free water. The PCR reaction was then run on the CFX96 real-time PCR system (BioRad, Hercules, CA, USA) with the cycling conditions of initial denaturation at 95 °C for 15 min, followed by 50 amplification cycles of denaturation at 95 °C for 30 s, annealing at 60 °C for 60 s, and extension at 75 °C for 30 s. The conditions for melting curve analyses were started from 55 °C to 85 °C with a ramp of 0.5 °C per 5 s. The amplification data were acquired in a real-time manner, and the melting temperatures of amplicons were analyzed following the amplification. The interpretation of results was automatically performed by Seegene Viewer Software, version 2.0 (version 2.0, Seegene Inc., Seoul, Republic of Korea), using pre-defined thresholds according to the manufacturer's recommendation. The positive and negative controls, as provided in the kit, were included in all runs, and the internal control was available in the master mix to detect possible inhibition in each reaction.

### DNA sequencing of *rpoB*, *katG*, and *inhA*

PCR amplification of specific portions of *rpoB, katG,* and *inhA* genes and DNA sequencing were performed as previously described^28^. For RIF resistance, a 278-bp fragment of the *rpoB* gene was amplified and sequenced (target positions 1519–1599) using primers *rpoB*-F (5-CAGGACGTGGAGGCGATCAC-3) and *rpo*B-R (5-GAGCCGATCAGACCGATGTTGG-3). For INH resistance, a 392-bp fragment of the *katG* gene was amplified and sequenced (target positions 823–1140) using primers *katG*-F (5-ATGGCCATGAACGACGTCGAAAC-3) and *katG*-R (5-CGCAGCGAG AGGTCAGTGGCCAG -3), and a 231-bp fragment of the *inhA* promoter was amplified and sequenced (target positions –1 to –50) using primers *inhA*-F (5-TCACACCGACAAACGTCACGAGC-3) and *inhA*-R (5-AGCCAGCCGCTGTGCG ATCGCCA-3). The cycling PCR amplification was performed as follows: initial denaturation at 96 °C for 5 min, followed by 35 cycles at 96 °C for 10 s, annealing at 55 °C for 10 s, and elongation at 72 °C for 30 s, and a cycle for a final extension at 72°C for 5 min. After amplification, the presence of PCR products was analyzed by agarose gel electrophoresis. Sequencing of PCR products was conducted using forward primers and the BigDye Terminator (version 3.1) sequencing kit, and carried out by an ABI PRISM 3130XL Genetic Analyzer (Applied Biosystems, Foster City, CA, USA). Mutations were analyzed from sequencing data by comparison with the *M. tuberculosis* H37Rv reference sequence using BioEdit software (version 7.1.10, Informer Technologies, Inc., Los Angeles, California, USA). Sequencing was used as a composite reference with phenotypic DST in this study.

### Data analysis

Data were described as frequencies or percentages when appropriate, with 95% confidence intervals (CIs). Using MGIT DST as a reference standard, sensitivity, specificity, positive predictive value (PPV), negative predictive value (NPV), and accuracy of the Anyplex MTB/MDR assay for the detection of INH-, RIF-resistance, MDR-TB, and drug-susceptible TB were determined using Medcalc (version 23.2.1, MedCalc Software Ltd., Ostend, Belgium) in calculation. The agreement of the Anyplex MTB/MDR assay with DNA sequencing was also examined.

## RESULTS

### Drug susceptibility and resistance identified by phenotypic DST

A total of 146 MTB isolates with phenotypic DST results were obtained and further analyzed in this study. Table 1 presents the drug-resistant profiles of all analyzed isolates. Of these, 50/146 (34.2%) were INH-mono-resistant isolates, 6/146 (4.1%) were RIF-mono-resistant isolates, 35/146 (24.0%) were INH- and RIF-resistant isolates (MDR-TB), and 55/146 (37.7%) were isolates susceptible to INH and RIF (Table 1).

**Table 1:** Drug resistance profiles by phenotypic DST of *Mycobacterium tuberculosis* used in this study and the performance of the Anyplex MTB/MDR assay for detecting INH and RIF resistance

MTB isolates		Phenotypic DST						Performance of the Anyplex MTB/MDR assay				
(n = 146)		INHR	RIFR	MDR	SUS	Total		Sensitivity	Specificity	PPV	NPV	Accuracy
								[95% Confidence interval, CI] (n)				
**The Anyplex MTB/MDR assay**	Genotypic DST											
	INH^R^	**42**	0	1	2	**45**		84.0 %	96.9%	93.3%	92.1%	92.5%
								[70.9-92.8]	[91.1- 99.4]	[82.1-97.7]	[85.9-95.6]	[86.9-96.2]
								(42/50)	(93/96)	(42/45)	(93/101)	(135/146)
	RIF^R^	0	**5**	4	1	**10**		83.3%	96.4%	50.0%	99.3%	95.9%
								[35.9-99.6]	[91.9-98.8]	[28.2-71.7]	[95.8-99.9]	[91.3-98.5]
								(5/6)	(135/140)	(5/10)	(135/136)	(140/146)
	MDR	2	0	**30**	0	**32**		85.7%	98.2%	93.8%	95.6%	95.2%
								[69.7-95.2]	[93.6-99.8]	[79.1-98.4]	[90.6-98.0]	[90.4-98.1]
								(30/35)	(109/111)	(30/32)	(109/114)	(139/146)
	Susceptible	6	1	0	**52**	**59**		94.6%	92.3%	88.1%	96.6%	93.2%
								[84.9-98.9]	[84.8-96.9]	[78.4-93.8]	[90.3-98.8]	[87.8-96.7]
								(52/55)	(84/91)	(52/59)	(84/87)	(136/146)
	Total (%)	**50 (34.2)**	**6 (4.1)**	**35 (24.0)**	**55 (37.7)**	**146 (100)**						
	Overall sensitivity for any INH^R^=88.2% (n=75/85) Overall sensitivity for any RIF^R^=95.1% (n=39/41)							Overall specificity for any INH^R^=96.7% (n=59/61) Overall specificity for any RIF^R^=97.1% (n=102/105)				

INH^R^ = isoniazid resistance; RIF^R^ = rifampicin resistance; MDR = multidrug resistance; SUS = susceptible; PPV = positive predictive value; NPV = negative predictive value; n = number of isolates.

### Detection results of genetic DST using Anyplex MTB/MDR

**Figure 1:**
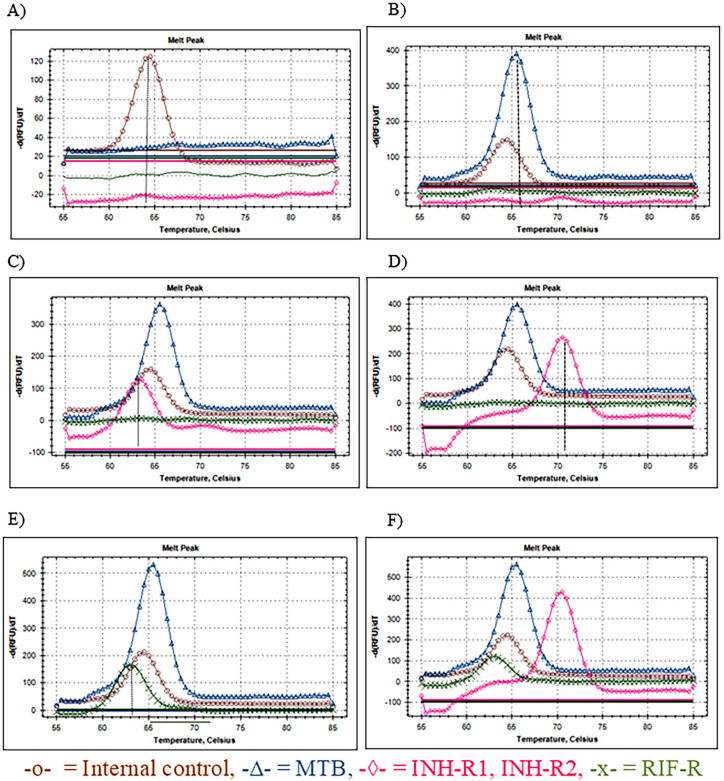
Examples of detection results by the Anyplex MTB/MDR real-time PCR. Plots show the rate of change of the relative fluorescence units with time (-d(RFU/dT) versus the temperature (Celcius). The representative melting curve profiles: (A) Negative control corresponds to *Tm* peak (in –o– line, brown peak) of internal control; (B) MTB corresponds to *Tm* peak (in –Δ– line, blue peak) of wild-type or mutant MTB; (C) High-level INH-mono-resistant MTB (INH-R1) corresponds to *Tm* peak (in –◊– line, pink peak) of *katG*-mutant MTB; (D) Low-level INH-mono resistant MTB (INH-R2) corresponds to *Tm* peak (in –◊– line) of mutant MTB with mutations in the *inhA* promoter); (E) Rifampicin-mono-resistant MTB (RIF-R) corresponds to *Tm* peak (in –x– line, green peak) of *rpoB*-mutant MTB; (F) Multidrug resistant MTB.

The assay was able to detect MTB and identify INH and RIF resistance in all 146 isolates within 3.5–4.5 h. The susceptibility and resistance to INH and RIF were identified based on the melting temperature analysis, and reported as MTB susceptible or resistant to INH and/or RIF without displaying the mutation patterns. Figure 1 depicts the representative melting peak profiles. For MTB detection, a melting peak at *Tm* ∼ 65.5 °C was observed using the FAM (6-carboxyfluorescein)–labeled probe. INH resistance was identified by a melting peak at *Tm* ∼ 64 °C for *katG* mutations or at *Tm* ∼ 71 °C for *inhA* promoter mutations, detected with the Cal Red 610–labeled probe. RIF resistance was indicated by a melting peak at *Tm* ∼ 64 °C for *rpoB* mutations using the HEX-labeled probe. The MDR isolate showed melting peaks corresponding to *rpoB, katG* or *inhA* mutations. Verification by the internal control demonstrated a melting peak at *Tm* ∼ 64.5 °C using the Quasar 670–labeled probe in the same reaction (Figure 1).

### Comparison of Anyplex MTB/MDR and phenotypic DST results

The results of genotypic DST using Anyplex MTB/MDR compared with the phenotypic DST are summarized in Table 1. The assay correctly detected 52/55 susceptible isolates. However, one susceptible isolate was detected as RIF-resistant and two as INH-resistant. Compared to the phenotypic DST, Anyplex MTB/MDR exhibited sensitivity of 84.0% (42/50), 83.3% (5/6), 85.7% (30/35), and 94.6% (52/55) for the detection of INH-mono-resistance, RIF-mono-resistance, MDR-TB, and drug-susceptible TB, respectively (Table 1). Overall, sensitivity of the Anyplex MTB/MDR assay for the detection of any INH resistance and any RIF resistance was 88.2% (75/85) and 95.1% (39/41), respectively. Regarding specificity, the assay showed 96.9% (93/96), 96.4% (135/140), 98.2% (109/111), and 92.3% (84/91) specificity for the detection of INH-mono-resistance, RIF-mono-resistance, MDR-TB, and drug-susceptible TB, respectively. Overall, specificity of the Anyplex MTB/MDR assay for the detection of any INH resistance and any RIF resistance was 96.7% (59/61) and 97.1% (102/105), respectively. The PPV was 93.3% (42/45), 50.0% (5/10), 93.8% (30/32) and 88.1% (52/59); the NPV was 92.1% (93/101), 99.3% (135/136), 95.6% (109/114), and 96.6% (84/87), for the detection of INH-mono-resistance, RIF-mono-resistance, MDR-TB detection, and drug-susceptible TB, respectively. The accuracy of the test was greater than 92.0% in overall testing (Table 1).

### Comparing the results of Anyplex MTB/MDR, DNA sequencing, and phenotypic DST

DNA Sequencing of the *rpoB*, and *katG* genes, and *inhA* promoter were performed in all 146 isolates. Various mutation types associated with INH, and RIF resistance were observed (Table 2). The distributions of all mutations (13 types: four in *katG*, one in *inhA*, and eight in *rpoB*) in this MTB population are shown in Table 2. All drug-resistant isolates exhibited a single mutation for resistance to each drug. A high agreement (> 95%) between DNA sequencing and Anyplex MTB/MDR was demonstrated (Table 2).

**Table 2:** Mutation analysis in *katG*, *inhA* and *rpoB* by sequencing in comparison to Anyplex MTB/MDR and phenotypic DST

Genotypic methods (n=146)							Nucleotide changes in detected mutations
DNA sequencing						Anyplex MTB/MDR	
**Phenotypic INH-R (n=50)**							*katG* mutation: 1) S315T (AGC>ACC) 2) S315N (AGC>AAC) 3) Y337C (TAC>TGC) 4) Ins1003G *inhA* mutation: 1) C–15T *rpoB* mutation: 1) S531L (TCG>TTG) 2) D516V (GAC>GTC) 3) H526R (CAC>CGC) 4) H526Y (CAC>TAC) 5) H526D (CAC>GAC) 6) L533P (CTG>CCC) 7) Q513L (CAA>CTA) 8) D516Y (GAC>TAC)
***katG* and *inhA* sequencing (n)**			***rpoB* sequencing (n)**				
	*katG* (34)						
		S315T (33)	No mutation (32), D516V (1)			INH-R (31) MDR (1)	
		Y337C (A1010G) (1)	No mutation (1)			Susceptible (1)*	
	*inhA* promoter (12)						
		C–15T (12)	No mutation (11), L533P (1)			INH-R (11) MDR (1)	
	No mutations (4)		No mutation (4)			Susceptible (5)	
**Phenotypic RIF-R (n=6)**							
No mutations (6)			H526Y (3)	Q513L (1)	S531L (1)	RIF-R (5)	
			No mutation (1)			Susceptible (1)	
**Phenotypic MDR (n=35)**							
	*katG* (27)						
		S315T (24)	H526R (7) H526D (3)	S531L (5) H526Y (2)	D516V (5) D516Y (1)	MDR (23)	
			No mutation (1)			INH-R (1)	
		S315N (2)	S531L (1)	D516V (1)		MDR (2)	
		Ins1003G (1)	S531L (1)			RIF-R (1)*	
	*InhA* promoter (5)						
		C–15T (5)	S531L (2)	H526D (2)	D516V (1)	MDR (5)	
	No mutations (3)		S531L (2)	D516V (1)		RIF-R (3)	
**Phenotypic drug-susceptible (n=55)**							
	*katG* (1)						
		S315T (1)	No mutation (1)			INH-R (1)	
	*inhA* promoter (1)						
		C–15T (1)	No mutation (1)			INH-R (1)	
	No mutations (53)		L533P (1), No mutation (52)			RIF-R (1) Susceptible (52)	
**DNA sequencing** *katG* mutations=62 *inhA* promoter mutations=18 *rpoB* mutations=42				**Anyplex MTB/MDR** *katG* mutations detected=59 *inhA* promoter mutations detected=18 *rpoB* mutations detected=42		

* *kat* mutations not detected by Anyplex MTB/MDR=Y337C and Ins1003G; n = number of isolates; INH-R = isoniazid resistance; RIF-R = rifampicin resistance.

By sequencing, 78/85 (91.8%) INH-resistant isolates had mutations in either *katG* or *inhA*; the *katG* gene accounted for 71.8% (61/85) of the mutations, and the *inhA* regulatory region accounted for 20.0% (17/85), while *katG* and *inhA* mutations were not detected in 5 INH-resistant isolates. Of 80 mutant strains by sequencing, Anyplex MTB/MDR could detect mutations in *katG* or *inhA* promoter region in 77 isolates (77/80, 96.3%), and missed *katG* mutations in three isolates. By *katG* sequencing, the majority (96.8%, 60/62) of *katG* mutants had mutations in codon 315. Two types of mutations were identified in this codon: S315T and S315N. The most common *katG* mutation was S315T (93.6%, 58/62), which was predominant in both INH-resistant (n=33) and MDR (n=24) isolates. Meanwhile, S315T was detected by Anyplex MTB/MDR and sequencing in one phenotypically susceptible isolate (Tables 2 and 3). The other *katG* mutation was S315N, found only in two MDR isolates. Besides, other distinct *katG* mutations were A1010G (nucleotide change), identified in one INH-resistant isolate, and Ins1003G, identified in one MDR isolate each (Tables 2 and 3). Discrepant results between Anyplex MTB/MDR and *katG* sequencing were observed in three isolates (3/62) (Tables 2 and 3). Overall, the high agreement between the Anyplex MTB/MDR assay and *katG* sequencing was demonstrated at 95.2% (59/62) (Table 2).

**Table 3:** Discordances in the detection of INH and RIF resistance by Anyplex MTB/MDR, DNA sequencing, and phenotypic DST

Phenotypic characteristics (n)		Results of	Anyplex MTB/MDR				DNA sequencing			Mutations
			INH		RIF		INH		RIF	
			*katG*	*inhA*	*rpoB*		*katG*	*inhA*	*rpoB*	
**Isoniazid-resistant MTB (8)**										
1	INH^R^-1		S	R	R		S	R	R	*inhA* C–15T, *rpoB* L533P (CTG>CCG)
2	INH^R^-2		R	S	R		R	S	R	*katG* S315T (AGC>ACC), *rpoB* D516V (GAC>GTC)
3	INH^R^-3		**S**	S	S		R	S	S	*katG* S315T (AGC>ACC)
4	INH^R^-4		**S**	S	S		**R**	S	S	*katG* Y337C (A1010G) (TAC>TGC)
5	INH^R^-5		S	S	S		S	S	S	None
6	INH^R^-6		S	S	S		S	S	S	None
7	INH^R^-7		S	S	S		S	S	S	None
8	INH^R^-8		S	S	S		S	S	S	None
**Rifampicin-resistant MTB (1)**										
	RIF^R^-1		S	S	S		S	S	S	None
**Multidrug-resistant MTB (5)**										
1	MDR-1		S	S	R		S	S	R	*rpoB* S531L (TCG>TTG)
2	MDR-2		S	S	R		S	S	R	*rpoB* D516V (GAC>GTC)
3	MDR-3		S	S	R		S	S	R	*rpoB* S531L (TCG>TTG)
4	MDR-4		**S**	S	R		**R**	S	R	*katG* InsG1003, *rpoB* S531L (TCG>TTG)
5	MDR-5		R	S	S		R	S	S	*katG* S315T (AGC>ACC)
**Susceptible MTB (3)**										
1	INH^S^ & RIF^S^-1 (SUS-1)		S	S	R		S	S	R	*rpoB* L533P (CTG>CCG)
2	INH^S^ & RIF^S^-2 (SUS-2)		R	S	S		R	S	S	*katG* S315T (AGC>ACC)
3	INH^S^ & RIF^S^-3 (SUS-3)		S	R	S		S	R	S	*inhA* C–15T

Number of discordant results of Anyplex MTB/MDR with phenotypic DST=17; Number of discordant results of Anyplex MTB/MDR with DNA sequencing=3 (underlined and bold); Number of discordant results of DNA sequencing with phenotypic DST=14; INH^R^ = isoniazid resistance; RIF^R^ = rifampicin resistance; MDR = multidrug resistance; S = susceptible; R = resistance; n = number of isolates; MTB =*Mycobacterium tuberculosis*; INH = isoniazid; RIF = rifampicin.

By sequencing, mutations in *inhA* promoter could be identified in 18 isolates (12 in INH-mono-resistant, 5 in MDR, and 1 in INH-susceptible isolates) (Table 2). Of these, only C−15T substitution was observed. No discrepant results were identified between Anyplex MTB/MDR and *inhA* sequencing, indicating perfect agreement between these methods.

By *rpoB* sequencing, mutations in the RRDR were detected in 42 isolates. Of these, 39 were phenotypically RIF-resistant MTB (95.1%). Eight different patterns of *rpoB* mutations were distributed among codons 531, 516, 526, 533 and 513 (Table 2). S531L mutation was the most common (12/41, 29.3%), followed by D516V (9/41, 21.0%) (Table 2). Regarding discordance, the three false-positive results detected as RIF-mutant MTB by the Anyplex MTB/MDR were identified. One was a susceptible isolate, which had L533P mutation (SUS-1) (Table 3). Another was an INH-mono-resistant isolate (INH^R^-1) and had the same L533P mutation. The other was an INH-mono-resistant isolate (INH^R^-2) and contained D516V mutation. All these mutations detected by Anyplex MTB/MDR were in agreement with sequencing (Tables 2 and 3). Regarding the two false-negative results detected by Anyplex MTB/MDR, one RIF-mono-resistant isolate (RIF^R^-1) showed no mutation in the *rpoB* gene and was identified as susceptible by Anyplex MTB/MDR and sequencing (Tables 2 and 3). The other isolate, a MDR strain, also showed no *rpoB* mutations but harbored a *katG* mutation, and was identified as INH-resistant MTB by Anyplex MTB/MDR and sequencing (Tables 2 and 3). In contrast, 102 of 105 RIF-susceptible isolates (97.1%) showed no mutations in RRDR. According to the results shown, Anyplex MTB/MDR and sequencing were concordant for all 42 isolates. However, 3 isolates (SUS-1, INH^R^-1, INH^R^-2) with *rpoB* mutations identified by these methods were RIF susceptible by phenotypic DST. Table 3 summarizes all discordant results based on the detection of mutations in *rpoB, katG,* and the *inhA* promoter by Anyplex MTB/MDR and sequencing compared with phenotypic DST.

## DISCUSSION

DR-TB remains a key factor in TB treatment failure and mortality. In high-burden countries like Thailand, where MDR-TB poses a significant public health challenge, phenotypic DST is critical for screening resistance to INH and RIF. Although phenotypic DST is considered a reference standard, its prolonged turnaround time is a major disadvantage, which can delay the initiation of effective therapy. Studies in Thailand and elsewhere have highlighted the necessity of genotypic DST. While rapid genotypic DST is increasingly used, phenotypic DST remains essential for confirming resistance and validating results, particularly in complicated cases like retreatment TB patients^29^. The concordance between the two tests has been documented, demonstrating reliability for identifying INH and RIF resistance^29,30^. The discordance is usually observed, particularly when resistance or mutations are not recognized by genetic tests, or phenotypic resistance is near the drug's critical concentration. A study in Thailand showed a low level of missing in the detection of INH resistance by the genotypic method, indicating the importance of phenotypic DST to detect resistance missed by genotypic tests^31^. Indeed, both genotypic and phenotypic methods provide useful information for TB and DR-TB treatment^32^. Combined assays as complementary testing should be performed^32^; phenotypic DST remains crucial when resistance is suspected but genotypic tests show susceptibility.

Early detection of INH and RIF resistance by genotypic DST can rapidly guide an appropriate regimen and assist in preventing TB treatment failure, including the progression to MDR-TB. This study demonstrates the good performance of Anyplex MTB/MDR for the rapid detection of INH and/or RIF resistance. Although genotypic DST can be impacted by the frequency and distribution of resistance-associated mutations in the microbial population^7,27,33^, diagnostic performance for Anyplex MTB/MDR remains high across studies. Our analysis showed similar results compared to previous studies reporting 83.3−100% sensitivity and 82.4−100% specificity for the detection of INH resistance^23,34,35^, 91.5−96.7% sensitivity and 98.1−100% specificity for detecting RIF resistance^34,35^, and 89.4% sensitivity and 100% specificity for detecting MDR-TB^35^. Meanwhile, Igarashi et al. reported a low sensitivity of 68.8% for the detection of INH resistance, and 93.8% sensitivity for detecting RIF resistance^36^. An evaluation study in Thailand utilizing various specimens in comparing Anyplex MTB/MDR with phenotypic DST, but not DNA sequencing, reported 85.71% sensitivity and 99.75% specificity for the detection of INH resistance, and 82.35% sensitivity and 99.75% specificity for detecting MDR-TB^25^. The difference across diagnostic values might be due to sample sizes, variations in the distribution of DR-TB, and mutations in microbial populations. Other independent factors might also affect the diagnostic performance values.

DNA sequencing identified mutations and their association with INH and RIF resistance. In addition, it served as a composite reference with the phenotypic DST, thereby providing more clarification on the discordance between the Anyplex MTB/MDR assay and phenotypic DST. It also provided valuable data on the frequency, and patterns of *katG, inhA,* and *rpoB* mutations. The findings of this present study show that the majority of mutations in this MTB population were common mutations in *katG, inhA,* and *rpoB*, suggesting that both INH and RIF resistance could be mainly identified by the detection of common mutations using genotypic DST. This study also highlights the need for local mutation data, which might show different genetic patterns of mutations associated with resistance to INH and RIF, some of which could affect the efficacy of detecting DR-TB using universal genotypic DST.

Variations in mutation frequency and distribution according to different geography and microbial populations have been reported^7,15,16^. In the present study, INH-resistant mutations were mainly found in *katG* at 71.8%, very similar to a previous study in Thailand reporting 72.1%^31^. The most common mutation in the *katG* gene was S315T (93.5%), prominent in both INH-mono-resistance and MDR-TB. Globally, about 64% of phenotypic resistance to INH can be attributed to the *katG* (S315T) mutation^7^. A less common *katG* mutation was S315N, which could be correctly identified by this real-time PCR assay in this study. Whereas, the only *inhA* mutation identified was C−15T, accounting for 20.0% of INH resistance in this population. It was reported that about 20–42% of INH-resistant MTB contained mutations in the *inhA* regulatory region, with the C−15T mutation being the most common^8^. The presence of C−15T alone in the *inhA* promoter region might be due to its high distribution. Approximately 80% of global INH-resistant isolates have been reported to contain mutations in codon 315 of the *katG* gene or −15 position in the *inhA* promoter^7^, while about 15% have unknown mechanisms^37^. Undetected mutations suggested the involvement of other genetic markers or various mechanisms of drug resistance. Regarding *rpoB* mutations, sequencing data showed that the most common mutations in the *rpoB* gene conferring RIF resistance were S531L, followed by D516V, and H526R. The high ratio of *rpoB* mutations (95.1%) existing in the RIF-resistant isolates indicates a strong association of *rpoB* mutations with RIF resistance as well as the high association of *katG* or *inhA* mutations (91.8%) with INH resistance. The high proportion of INH resistance caused by *katG* mutations compared to *inhA* mutations presumably resulted from the widespread transmission of the *katG*-mutant strains. In contrast, few distinct mutations were presented. These rare mutations were missed by Anyplex MTB/MDR, suggesting that low-frequency mutations or new mutation variants might cause limitations in genotypic DST.

Discordance in the detection of INH and RIF resistance by both molecular methods might be due to certain factors. Insufficient detection of INH or RIF resistance can miss hetero-resistance, which contains drug-resistant and susceptible bacilli^27,33^. Furthermore, Anyplex MTB/MDR is not able to detect resistance-conferring mutations outside the target regions, and other non-targeted mutations involved in resistance such as infrequent or rare mutations. Another possible reason for discrepancies is due to mis-detection by genotypic DST^7^. Other mechanisms of resistance to both RIF and INH may lead to discordant results in DST. Since INH and RIF resistance are known to involve various mechanisms, resistance to INH and RIF might be mediated by other processes that do not involve alterations in the DNA sequences, such as changes to cellular mechanisms or drug target overexpression^3,38^. Also, uncommon mutations were associated with a susceptible phenotype or low-level resistance to INH^8,37^. For RIF, some *rpoB* mutants contained borderline mutations which usually confer low-level RIF resistance, such as L533P and D516V^39^. Because the resistance is low-level or variable, it is frequently missed by phenotypic DST (MGIT-DST).

### Limitations

This present study is limited by the use of MTB cultured isolates rather than direct specimens, as well as by the small numbers of RIF-mono-resistant isolates. In addition, no genetic analyses of mutations in other genes that might confer resistance to INH have been performed. Further studies involving whole-genome sequencing (WGS) of each MTB drug-resistant strain would yield comprehensive information on mutations, including novel or rare mutations that other genotypic methods might misdetect. Despite the limitations, this study presents the potential for use of the Anyplex MTB/MDR assay for the rapid detection of INH and RIF resistance. The high frequency of common mutations found suggests that genotypic DST would perform well in this setting.

## CONCLUSIONS

The Anyplex MTB/MDR assay shows promising performance for the simultaneous identification of MTB, INH, and RIF resistance due to its high sensitivity, specificity, PPV, NPV, and accuracy compared to phenotypic DST, with strong agreement in comparison to DNA sequencing. The assay has the potential to be used as a rapid and accurate DST for guiding appropriate and timely treatment of TB patients. Various mutations were present in this population, with a high degree of association between *katG* and *inhA* mutations and INH resistance, and between *rpoB* mutations and RIF resistance, the most prevalent being S315T in *katG*, C−15T in *inhA*, and S531L followed by D516V in *rpoB*. The borderline *rpoB* was observed. This information is expected to be useful for guiding the use and further improvements of molecular tests for the detection of TB and resistance to INH and RIF.

## Data Availability

The anonymized dataset generated during this study is available from the corresponding author upon reasonable request.
